# Evaluation of the role of staphylococci in the pathomechanism of conjunctivitis

**DOI:** 10.1007/s10792-021-01818-w

**Published:** 2021-03-28

**Authors:** Ewa Jasińska, Agnieszka Bogut, Agnieszka Magryś, Alina Olender

**Affiliations:** 1 Department of General Ophthalmology, Medical University of Lublin, Lublin, Poland; 2 Chair and Department of Medical Microbiology, Medical University of Lublin, Lublin, Poland

**Keywords:** *Staphylococcus*, Conjunctivitis, Biofilm, Adhesion, Cytotoxicity assay, Drug-resistance

## Abstract

**Purpose:**

Determination of the association between *ica* genes and phenotypic biofilm formation in staphylococcal isolates involved in conjunctivitis, their antibiotic resistance as well as detection of selected virulence characteristics: adhesion to epithelial cells and in vitro cytotoxicity.

**Methods:**

The study included 26 *Staphylococcus aureus* (SA) and 26 *Staphylococcus epidermidis* (SE) isolates. The presence of *icaAD* genes and *ica* operon was determined by the PCR assay. Phenotypic biofilm formation was verified using the microtitre plate assay. Antibiotic resistance was performed using the disc diffusion method. Staphylococcal ability to attach to host cells was assessed by flow cytometry. Cytotoxicity on epithelial cells was evaluated by LDH assay.

**Results:**

The *ica* genes were detected in 26.9% of SE and in 42.3% of SA isolates. Only 15.3% of isolates (SE) were positive for both the *icaAD* and the *ica* operon. Phenotypically, 19.2% of SE isolates were strong biofilm producers, among which three were both *icaAD*- and *ica* operon-positive. About 26.9% of SA isolates were strong biofilm producers. Methicillin resistance (MR) was detected in 34.6% of SE and 26.9% of SA isolates. About 75% of MR isolates were multidrug resistant. SA isolates adhered to host cells more extensively than SE. SA isolates released higher level of LDH than SE.

**Conclusions:**

Adherence abilities were commonly observed in staphylococci associated with conjunctivitis. However, low prevalence of isolates positive for a complete and functional *ica* locus and low prevalence of strong biofilm producers was detected. SA adhered to a greater extent to eukaryotic cells than SE and were more cytotoxic.

## Introduction

Staphylococcal species including *Staphylococcus aureus* (SA) and *Staphylococcus epidermidis* (SE) are among the most common bacterial pathogens involved in the aetiology of acute conjunctivitis [1].

Important aspects of pathogenic potential of staphylococci linked to their ability to cause conjunctivitis include initial adherence, inherent virulence, cytotoxicity, as well as evasion of the host immune system and antimicrobial tolerance/resistance facilitated by the biofilm production [2–4].

Adherence to host cells is a preliminary step necessary to initiate and establish infection. Staphylococci express a series of proteins/adhesins, such as the microbial surface components recognizing adhesive matrix molecules (MSCRAMMs) that bind fibrinogen, fibronectin, collagen, laminin and play a prominent role in the initial adhesion [5–7]. Once attachment to tissue is accomplished, staphylococcal biofilm is able to grow by proliferation and production of a scaffolding extracellular matrix. The important matrix components in the staphylococcal biofilm include the polysaccharide intercellular adhesin (PIA), extracellular DNA (eDNA), teichoic acids, and proteins [8, 9].

The PIA consisting of the poly-β(1–6)-N-acetylglucosamine and encoded by the *ica* operon is regarded essential for the biofilm formation. In addition to bacterial aggregation and important structural functions in the biofilm matrix architecture and is implicated in bacterial adhesion to biomaterial surfaces as well as evasion from the host immune response. The *ica* locus consists of the *icaADBC* operon that contains four genes encoding proteins required for the generation of the PIA. The *icaA* and *icaD* are considered the principal genes involved in the synthesis of the exopolysaccharide. The product of the *icaA* gene is a transmembrane protein with N-acetylglucosaminyl transferase activity necessary for the synthesis of the poly-N-acetylglucosamine polymer. Protein encoded by the *icaD* gene, in turn, is considered essential for the most favourable enzymatic activity of the *icaA* gene product. Enzymatic activity of the *icaA gene* product becomes significant and olygomers longer than 20 residues are synthesized only when coexpressed with the *icaD* gene product [10].

Although PIA represents a major mechanism of the biofilm production both in SA and in SE, certain staphylococcal surface proteins can also promote the accumulation phase by the *ica*-independent mechanism. The cell wall anchored proteins mediate primary attachment but also promote intercellular adhesion, biofilm accumulation and maturation. This is subsequently followed by dispersal phase during which the biofilm structure is disrupted by enzymatic degradation of matrix components by proteases, nucleases, and a group of α-helical peptides called phenol-soluble modulins (PSMs) functioning as surfactants [8, 10].

Although staphylococci are generally classified as extracellular bacteria, recent data revealed that these bacteria can invade various types of host cells, professional phagocytes and non-phagocytic cells, including fibroblasts, epithelial cells and others. Internalized bacteria can either induce host cell apoptosis or survive and persist intracellularly for several days [5, 7]. The induction of host cell death has been linked with the ability of SA to secrete cytolytic toxins, such as α-toxin or Panton–Valentine leucocidin (PVL) [6]. Unlike SA, which typically produces aggressive virulence determinants, commensal SE seems to possess a limited number of virulence factors, which rather promote its persistence, not invasive infections [11, 12].

The aim of the study was determination of the association of the *ica* genes with the phenotypic biofilm formation in staphylococcal isolates cultured from patients with conjunctivitis, analysis of the association regarding the *ica* gene detection, phenotypic biofilm production capacity and the antibiotic resistance profiles of the isolates as well as detection of selected virulence characteristics associated with adhesion to epithelial cells and cytotoxicity in the in vitro model (cell line Detroit 562).

## Materials and methods

### Bacterial strains

The study included a total of 52 staphylococcal isolates (26 SA isolates and 26 SE isolates) cultured from 52 conjunctival swabs collected from patients demonstrating the clinical signs and symptoms of bacterial conjunctivitis. Each conjunctival swab was obtained from the lower fornix of each case and subsequently cultured onto the sheep blood agar and mannitol salt agar (MSA). The inoculated media were incubated at 37 °C for up to 48 h. The identification of isolates growing in cultures was initiated with the use of conventional laboratory methods including Gram staining, catalase reaction, haemolytic activity on the sheep blood agar, growth on the mannitol salt agar (MSA), as well as the coagulase test. Final identification of the isolates to the species level was performed using the ID32 STAPH (BioMérieux) biochemical test according to the manufacturer’s instructions.

### Antimicrobial susceptibility

Susceptibility of staphylococcal isolates to antimicrobial drugs was evaluated using the disc diffusion method. The tests were performed and interpreted according to the current recommendations of the European Committee on Antimicrobial Susceptibility Testing (EUCAST). The tested antibiotics included the following: cefoxitin (FOX), erythromycin (E), clindamycin (CC), tetracycline (TE), norfloxacin (NOR), ciprofloxacin (CIP), ofloxacin (OFX), levofloxacin (LVX), moxifloxacin (MXF), gentamicin (GM), amikacin (AN), neomycin (N), chloramphenicol (C), and co-trimoxazole (TMP/SXT).

## PCR detection of the *ica* genes

### Bacterial DNA extraction

Bacterial DNA for the PCR detection of the *ica* genes was isolated using the following procedure: staphylococcal colonies grown on the blood agar medium were collected with an inoculation loop and suspended in 150 µl of water. The solution was incubated at 95 °C for 10 min followed by 10 min incubation in an ultrasonic bath. The solution was then centrifuged for 5 min at a maximum speed, and the supernatant containing the DNA was transferred to a new tube and subsequently used for the PCR reaction.

### PCR method for amplification of *icaA* and *icaD* genes

The primers were synthesized by Genomed (Poland).

For the detection of *icaA*, 5'-TCTCTTGCAGGAGCAATCAA was used as the forward primer, and 5'-TCAGGCACTAACATCCAGCA was used as the reverse primer [9]. The two primers enabled to obtain a 188-bp amplification product.

For the detection of *icaD*, 5'-ATGGTCAAGCCCAGACAGAG was used as the forward primer, and 5'-CGTGTTTTCAACATTTAATGCAA was used as the reverse primer [9]. The two primers enabled to obtain a 198-bp amplification product.

The PCR reaction was conducted in a 50 µl volume containing the above-mentioned primers (1 µmol each), together with the extracted bacterial DNA, 0.2 mM of each of dATP, dCTP, dGTP, dDTP, 1.25 U Dream Taq Polymerase, and 10X Dream Taq Buffer (Thermo Scientific).

A thermal profile of the reaction was as follows: incubation at 95 °C for 2 min, followed by 35 cycles at 95 °C for 30 s (denaturation), 55 °C for 30 s (annealing), 72 °C for 1 min. (extension), and 72 °C for 10 min. (final extension) after the 35 cycles.

After amplification, 10 µl of the PCR mixture was analysed by agarose gel electrophoresis (2% agarose in Tris–borate–EDTA). Molecular weight marker ФX174 DNA/BsuRI (HaeIII) Marker (Thermo Scientific) was used.

### PCR method for amplification of *ica* operon

The primers were synthesized by Genomed (Poland).

The presence of the entire *ica* operon was verified in SA and SE strains by the amplification of a 2.7 kb gene product encompassing a region of the *icaADBC* locus [13].

For the assay, 5′-TGCACTCAATGAGGGAATCA was used as the forward primer, and 5′-AATCACTACCGGAAACAGCG was used as the reverse primer [13].

The PCR reaction was conducted in a 50 µl volume containing the above-mentioned primers (1 µmol each), together with the extracted bacterial DNA, 0.2 mM of each of dATP, dCTP, dGTP, dDTP, 1.25 U Dream Taq Polymerase, and 10X Dream Taq Buffer (Thermo Scientific).

A thermal profile of the reaction was as follows: incubation at 95 °C for 2 min, followed by 35 cycles at 95 °C for 30 s (denaturation), 55 °C for 30 s (annealing), 72 °C for 2 min. (extension), and 72 °C for 10 min. (final extension) after the 35 cycles.

After amplification, 10 µl of the PCR mixture was analysed by agarose gel electrophoresis (2% agarose in Tris–borate–EDTA). Molecular weight DNA Marker 3 (A&A Biotechnology, Poland) was used.

### Phenotypic characterization of the biofilm-producing ability

Quantitative determination of biofilm production was performed with the use a microtitre plate assay (MPA) with crystal violet. Briefly, the overnight bacterial culture of each tested bacterial isolate was adjusted with tryptic soy broth (TSB, BioMérieux) to match the turbidity of 0.5 McFarland standard. The suspension was subsequently incubated overnight at 37 °C. The solution was diluted 1:100 in TSB and 200 µl aliquots were inoculated into three wells each of the 96-well sterile microtitre plate. The plates were incubated overnight at 37 °C in air, washed, and stained with 0.1% crystal violet. The optical density was measured at 570 nm. The following values of absorbance were used for the interpretation of the biofilm forming capacity [14]:

-  ≤ 0.120: the isolate classified as nonadherent (biofilm-negative)
-  > 0.120 ≤ 0.240: the isolate classified as weakly adherent
-  > 0.240: the isolate classified as strongly adherent

A reference strain of *S. epidermidis* ATCC 35984 was used as a positive control.

### Cell line culture and preparation of bacteria for in vitro experiments

Detroit 562 (CCL-138, ATCC), a human epithelial cell line derived from pharyngeal carcinoma, was maintained in a continuous culture in DMEM medium (Corning) containing 10% heat-inactivated foetal bovine serum (FBS; SIGMA Aldrich) at 5% CO_2_ at 37 °C.

For in vitro experiments, SE (*n* = 26) and SA (*n* = 26) strains were used. Bacterial strains were incubated to mid log phase (OD600 = 0.6) in 5 ml of tryptic soy broth (TSB; Oxoid) at 37 °C under constant rotation. The cells were then harvested by centrifugation (2.500 × g for 5 min) and washed twice in PBS. Bacterial chains and aggregates were broken by mild sonication for 3 × 10 s. at 30 W (Bransonic ultrasonic cleaner; G. Heinemann) on ice. Samples were then centrifuged (5.000 × g for 10 min), and the pellets were resuspended in DMEM. The accuracy of preparation of bacterial samples was routinely verified by plating dilutions on agar plates and counting colonies to determine colony forming units (CFU) per ml.

### Internalization assay

For internalization assay, Detroit 562 cells (3 × 10^5^ cells/well) were plated in a 24-well culture plate and infected by bacteria at MOI of 30. Detroit cells and staphylococci were cocultured for 2 h in humidified atmosphere containing 5% CO_2_. Internalization was stopped by placing the plate on ice and washing the cells twice with ice-cold PBS to remove non-internalized bacteria. The culture medium was replaced by DMEM with 10% FBS containing gentamicin (100 μg/ml) + vancomycin overnight. After the incubation, the cells were washed with PBS and the medium was changed again to fresh media without antibiotics, and the culture was intended for determination of bacterial cytotoxicity assay.

### Determination of cytotoxicity using the lactate dehydrogenase [LDH] release assay

To determine the cytotoxic properties of the tested bacterial strains in cultured Detroit 562 cells, the commercially available Pierce LDH Cytotoxicity Assay Kit (Thermo Scientific) was used, following the manufacturer's instructions. The specific cytotoxicity was calculated using the following formula:

% cytotoxicity = (tested LDH activity—control LDH activity)/(maximum LDH activity—spontaneous LDH activity) × 100.

Relative amounts of LDH release were measured (absorbance at 490 nm) using an ELISA plate reader. The cytotoxic activity of tested staphylococcal strains was determined 24 h after internalization and expressed as the percentage of killed cells. All assays were performed in three independent replications.

### Staphylococcal attachment to Detroit 562 cell line as assessed by flow cytometry (adhesion assay)

Detroit 562 cells were seeded at 2 × 10^5^ cells per well onto 24-well tissue culture plates in 1 mL of DMEM medium and cultured at 37 °C in 5% C0_2_ for 1–2 days until 80–90% confluency. The viability of the Detroit was confirmed by staining with 0.2% w/v trypan blue (Sigma Aldrich).

Bacteria labelled with BODIPY-FL (Molecular Probes) at MOI 40 were inoculated with the cells for 15 min at 37 °C in 5% CO2. To stop the internalization, plates were placed on ice for 5 min. Non-adherent staphylococci were removed by washing the cells three times with ice-cold PBS. After detaching the Detroit cells, they were transferred to clean tubes and centrifuged (200 × g, 5 min., 4 °C). The cell pellets were resuspended in one mL of cold DMEM culture medium and then processed through the flow cytometry. Bacteria attached to Detroit were quantified by a flow cytometer BD FACSCCalibur (BD Bioscience) using BD CellQuest software (BD Bioscience). A minimum of 50,000 cells was measured in each sample. This experiment was repeated three times for each strain.

### Statistical analysis

Data are expressed as means ± SD of a minimum of two independent experiments. Data were assessed with non-parametric Mann–Whitney test (for independent variables) to compare the differences between the two types of staphylococcus. Non-parametric chi-square test (for qualitative variables) was used to compare the frequency of the analysed categories depending on the type of staphylococcus. Spearman correlation coefficient test was used to determine the relationship between the examined features. P values of < 0.05 were considered to be statistically significant.

## Results

### The *ica* genes detection by PCR and its correlation to the phenotypic biofilm production

Overall, the *ica* genes were detected by PCR in seven (26.9%) out of 26 SE isolates and in 11 (42.3%) out of 26 SA isolates included in the study.

**Fig. 1:**
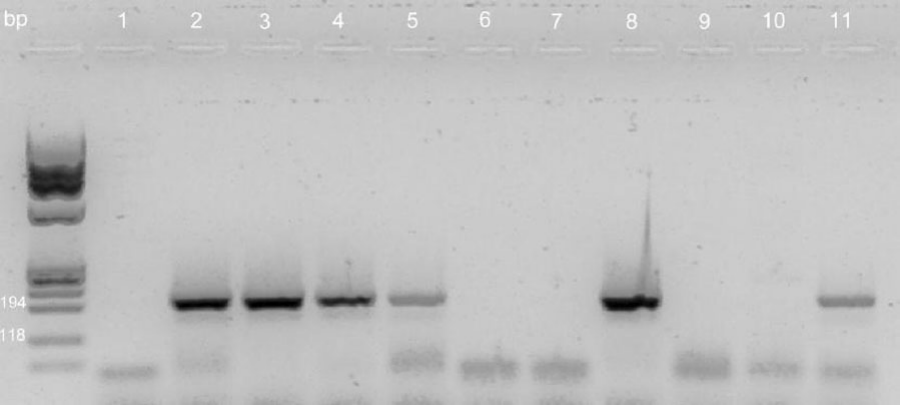
PCR detection of the icaA and icaD genes in SA and SE isolates. Lane 1, absence of amplification product from a strong SE biofilm producer (31 III SE); Lane 2, icaA-positive, strong SE biofilm producer (21 SE); Lane 3, icaD-positive, strong SE biofilm producer (21 SE); Lane 4, icaA-positive, weak SA biofilm producer (40 SE); Lane 5, icaD-positive, weak SA biofilm producer (40 SE); Lanes 6–7, icaA- and icaD-negative (respectively) strong SA biofilm producer (54 SA); Lane 8, icaD-positive, weak SA biofilm producer (25 SA); Lanes 9–10, icaA- and icaD-negative (respectively) weak SE biofilm producer (42 SE); Lane 11, icaA-positive, weak SE biofilm producer (13 SE);

**Fig. 2:**
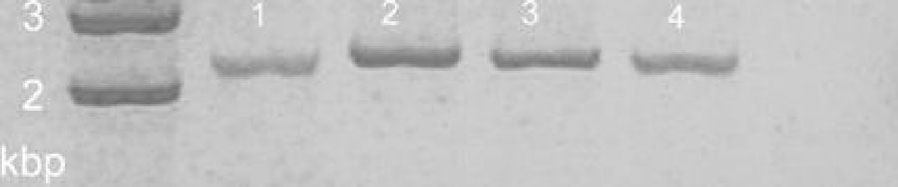
PCR detection of the ica locus in SE isolates. Lanes 1–3, ica locus-positive, strong SE biofilm producers (21 SE, 31 III SE, 35 SE); Lane 4, ica locus-positive, weak SE biofilm producer (31 SE)

Nevertheless, we detected high diversity of the detected *ica* profiles (Figs. 1, 2). Only, a minority of the isolates (represented only by SE) were positive for both the *icaAD* and the *ica* operon which was indicative of a complete set of genes required of the PIA synthesis.

Overall, the *ica* profiles detected by PCR included the *icaD* (10/38.4% SA isolates), *icaAD* + *ica* operon (4/15.3% SE isolates), *icaAD* (1/3.8% SA isolate and 2/7.6% SE isolates), and *icaA* (1/3.8% SE isolate). The results of the PCR *ica* detection in all isolates included in the study are demonstrated in Table. 1.

**Table 1:** Characterization of staphylococcal isolates associated with conjunctivitis: ica gene detection profiles, phenotypic biofilm production, and antibiotic resistance results

No	Isolate no	Antibiotic resistance profile	Mean absorbance value (OD570)/ biofilm production capability**	*ica* genes detected by PCR
*Staphylococcus epidermidis* isolates				
1	2 SE	Fully susceptible	0.135/weak	–
2	7 SE	TE	0.143/weak	–
3	12 SE	Fully susceptible	0.149/weak	–
4	13 SE	SXT, OFX	0.150/weak	icaA
5	17 SE	MLS_B_	0.142/weak	–
6	18 SE	Fully susceptible	0.144/weak	
7	19 SE	Fully susceptible	0.148/weak	
8	20 SE	MLS_B_	0.173/weak	–
9	21 SE*	FOX, TE, C	0.599/strong	icaAD,
ica operon				
10	23 SE*	FOX, MLS_B_, TE, NOR, CIP, OFX, MXF, SXT	0.187/weak	icaAD
11	28 SE*	FOX, MLS_B_, NOR, CIP, OFX, LVX, MFX, GM, AN, N	0.175/weak	icaAD
12	31 SE*	FOX, MLS_B_, NOR, CIP, OFX, LVX, MFX, GM, C	0.161/weak	icaAD,
ica operon				
13	31 I SE	FOX, MLS_B_	0.142/weak	–
14	31 III SE	MLS_B_	0.645/strong	icaAD,
ica operon				
15	31VII SE	MLS_B_	0.252/strong	–
16	31VIII SE	FOX	0.166/weak	–
17	A SE	MLS_B_	0.149/weak	–
18	33 SE	Fully susceptible	0.135/weak	–
19	34 SE*	MLS_B_, TE, CIP, OFX, LFX, GM, AN, N, SXT	0.339/strong	–
20	35 SE*	MLS_B_, TE, OFX, SXT	0.552/strong	icaAD,
ica operon				
21	36 SE*	FOX, MLS_B_, SXT	0.136/weak	–
22	39 SE	TE	0.151/weak	–
23	42 SE*	FOX, MLS_B_, TE, NOR, CIP, OFX, LVX, MFX, GM, N, SXT	0.184/weak	–
24	45 SE	FOX, N	0.193/weak	–
25	46 SE	MLS_B_	0.129/weak	–
26	49 SE	MLS_B_	0.128/weak	–
*Staphylococcus aureus* isolates				
1	1SA	Fully susceptible	0.169/weak	ica D
2	3 SA	Fully susceptible	0.179/weak	ica D
3	4 SA	TE	0.162/weak	–
4	5 SA	Fully susceptible	0.348/strong	–
5	6 SA	Fully susceptible	0.157/weak	–
6	8 SA	TE, GM, N	0.189/weak	icaD
7	9 SA	Fully susceptible	0.162/weak	–
8	10 SA	Fully susceptible	0.153/weak	–
9	11 SA	Fully susceptible	0.337/strong	ica D
10	15 SA	Fully susceptible	0.273/strong	–
11	16 SA	Fully susceptible	0.206/weak	–
12	22 SA	Fully susceptible	0.200/weak	ica D
13	24 SA	Fully susceptible	0.205/weak	ica D
14	25 SA*	FOX, MLS_B_, NOR, CIP, C	0.212/weak	IcaD
15	30 SA*	FOX, MLS_B_, C	0.185/weak	IcaD
16	32 SA	Fully susceptible	0.264/strong	–
17	37 SA	Fully susceptible	0.204/weak	–
18	40 SA	Fully susceptible	0.158/weak	ica AD
19	47 SA	TE	0.468/strong	–
20	48 SA	MLS_B_	0.138/weak	–
21	50 SA*	FOX, MLS_B_, GM, AN, N	0.191/weak	icaD
22	53 SA	FOX, MLS_B_	0.186/weak	icaD
23	54 SA*	FOX, MLS_B_, NOR, CIP, OFX, LVX, MFX, AN, N	0.383/strong	–
24	56 SA*	FOX, MLS_B_, NOR, CIP, OFX, LVX, MFX, AN, N	0.328/strong	–
25	57 SA*	FOX, MLS_B_, NOR, CIP, OFX, LVX, MFX, AN, N	0.203/weak	–
26	58 SA	Fully susceptible	0.153/weak	–

^*^Multidrug resistance (MDR); **OD ≤ 0.120 – non-adherent; OD > 240 – strongly adherent; OD > 120 ≤ 0.240 – weakly adherent; SA—Staphylococcus aureus, SE—Staphylococcus epidermidis, MLS_B_—cross-resistance to macrolides, lincosamides, and streptogramin B, cefoxitin; detection of methicillin resistance (FOX), erythromycin (E), clindamycin (CC), tetracycline (TE), norfloxacin (NOR), ciprofloxacin (CIP), ofloxacin (OFX), levofloxacin (LVX), moxifloxacin (MXF), gentamicin (GM), amikacin (AN), neomycin (N), chloramphenicol (C), co-trimoxazole (TMP/SXT)

In spite of the low level of the *ica* detection in the analysed staphylococci, all tested isolates demonstrated adherence capabilities under applied in vitro conditions. Strong biofilm production identified on the basis of criteria adopted from Christensen et al. was detected in the cumulative number of 12 (23%) out of the 52 staphylococcal isolates (Christensen et al., [14]). The remaining isolates were classified as weak biofilm producers. The results of the phenotypic biofilm production using the MTP assay are demonstrated in Table 1.

Comparable numbers of SA (26.9%) and SE (19.2%) isolates were strong biofilm producers. Among five SE strong biofilm producers only three were both the *icaAD*- and *ica* operon-positive. The remaining proficient biofilm producers among SE isolates were *ica*-negative. Among seven SA strong biofilm producers only one was positive for the *icaD* gene. The remaining proficient biofilm producers among SA isolates were *ica*-negative.

The greatest biofilm biomass (OD > 0.5) was observed for three SE isolates positive for both the *ica* operon and *icaAD*. Nevertheless, in the group of *ica*AD- and *ica* operon-positive SE isolates, one isolate (no. 31) most probably did not produce a functional PIA under experimental conditions since it was classified as a weakly adherent isolate (OD = 0.161).

### Antibiotic resistance in correlation to the *ica* gene profiles and phenotypic biofilm production

Methicillin resistance (MR) was detected in 9 (34.6%) SE and 7 (26.9%) SA isolates (not significant). Twelve out of the 16 (75%) MR isolates were multidrug resistant/MDR (resistant to ≥ 3 antimicrobial groups).

MR was most frequently accompanied by resistance to macrolides, lincosamides and group B streptogramins (MLS_B_ phenotype)—detected in 12 out of the 16 MR (75%) isolates and fluoroquinolones—detected in 8 (50%) MR isolates. As many as six of the fluoroquinolone-resistant isolates were resistant to all tested agents belonging to this group of antimicrobials including NOR, CIP, OFX, MXF, and LVX. Aminoglycoside resistance was observed in 7 (43.7%) MR isolates with diverse resistance profiles: GM + N + AN- 2 isolates, GM-1 isolate, GM + N-1 isolate, AN + N- 3 isolates. Chloramphenicol, co-trimoxazole, and tetracycline resistance was detected in 4 (25%), 3 (18.7%), and 3 (18.7) MR isolates, respectively.

Methicillin susceptible isolates exhibited MLS_B_ resistance phenotype most frequently (10 out of 36 isolates/27.7%), followed by tetracycline resistance (6 isolates/16.6%).

We did not observe any correlation between methicillin resistance (MR) and the *ica* genes presence and/or the phenotypic biofilm production.

Strong biofilm production was observed for only three MR staphylococcal isolates (one SE and two SA) and only one of these isolates (SE no. 21) harboured the *icaAD* and *ica* operon correlating to the production of the functional PIA.

Moreover, among the 12 MR isolates defined as multidrug resistant, two MRSA isolates (no. 54, 56) were strongly adherent but *ica*-negative and one MRSE isolate (no. 21) was a strong biofilm producer positive for the *icaAD* and *ica* operon.

It should also be mentioned that strong biofilm production was detected in two other MDR, but methicillin-sensitive isolates represented by SE (no. 34 and 35). One of these isolates (no. 34) was *icaAD* and *ica* operon-positive.

Among the remaining 20 staphylococcal isolates fully susceptible to the tested antimicrobial agents (5 SE isolates and 15 SA isolates), four SA isolates were *icaD*-positive (and weakly adherent by the MTP assay) and 1 SA isolate was *icaAD*-positive (and weakly adherent by the MTP assay).

### Attachment of *S. epidermidis* and *S. aureus* to cultured epithelial cells

**Fig. 3:**
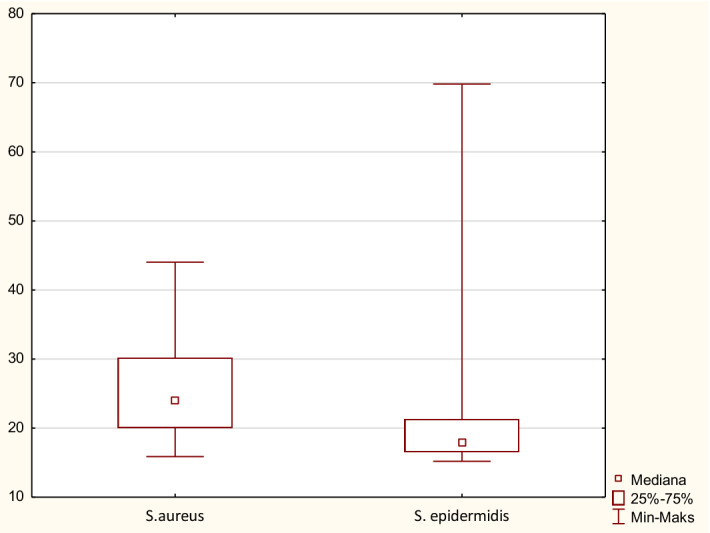
Assessment of the attachment value in *S. aureus* and *S. epidermidis* strains (pooled data)

**Fig. 4:**
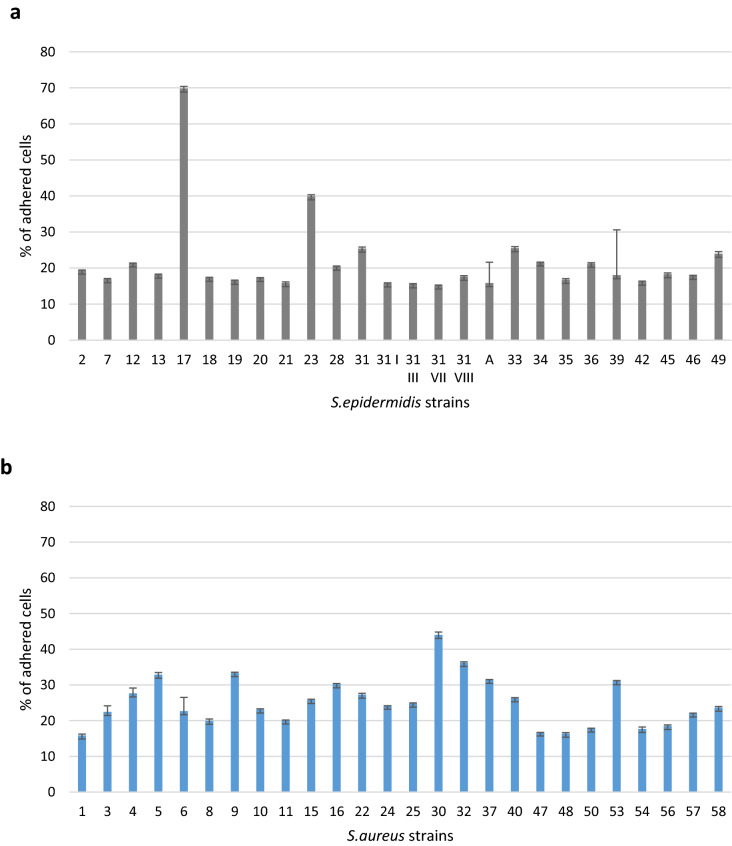
Attachment to Detroit 562 cells by different staphylococcal clinical isolates. **a** represents attachment ability of *S. epidermidis* strains. **b** represents attachment ability of *S. aureus* strains Results expressed as mean % of attached bacterial cells/50 000 epithelial cells ± SD from at least three independent experiments

Cells originating from nasopharynx were used as host cells to assess the capacity of SE (*n* = 26) and SA (*n* = 26) strains to attach. All tested bacterial strains, both *S. aureus* and *S. epidermidis* attached to Detroit 562 cell line after 15 min. contact. The ability of the tested strains to attach to the Detroit cells was variable, ranging from 15.8% (strain no. 1) to 44% (strain no. 30) for SA and 15.1% (strain no. 31 VII) to 69.8% (strain no. 17) for SE. *S. aureus* isolates however showed the statistically highest attachment capacity, while SE strains showed a lower attachment pattern (*p* < 0.05; Fig. 3). As presented on Fig. 4a, in 17 cases (65%) of SE strains, less than 20% of their cells attach to epithelial cells. Similarly, low attachment ability was observed only in 6 out of 26 (23%) SA strains (Fig. 4b).

In order to check whether bacterial ability to attach to epithelial cells corresponds with their ability to form biofilm, statistical analysis of correlation between these two traits of tested staphylococcal strains was performed. As a result, no correlation between attachment to host cells and biofilm formation was found. This finding indicates that different bacterial factors are involved in both determinants of pathogenicity of staphylococci.

### Lactate dehydrogenase (LDH) measurement—cytotoxic abilities of different staphylococcal clinical isolates

**Fig. 5:**
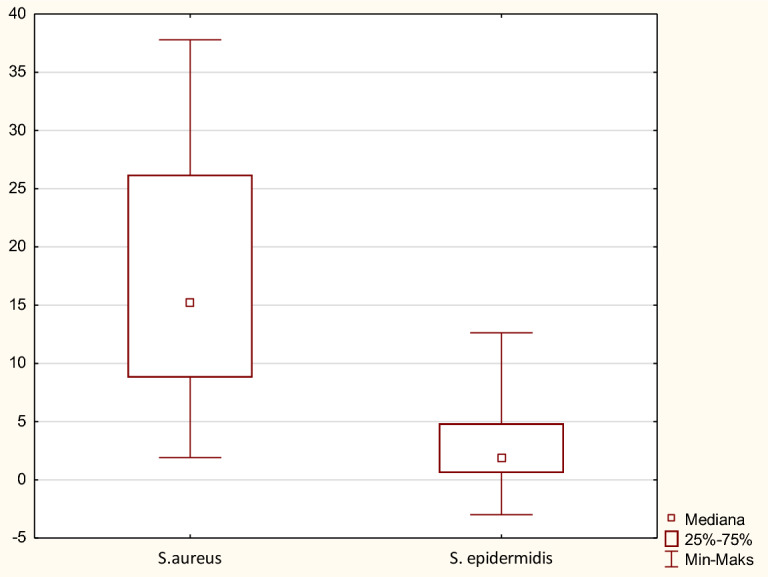
Cytotoxic properties of *Staphylococcus aureus* strains and *S. epidermidis* strains (pooled data)

**Fig. 6:**
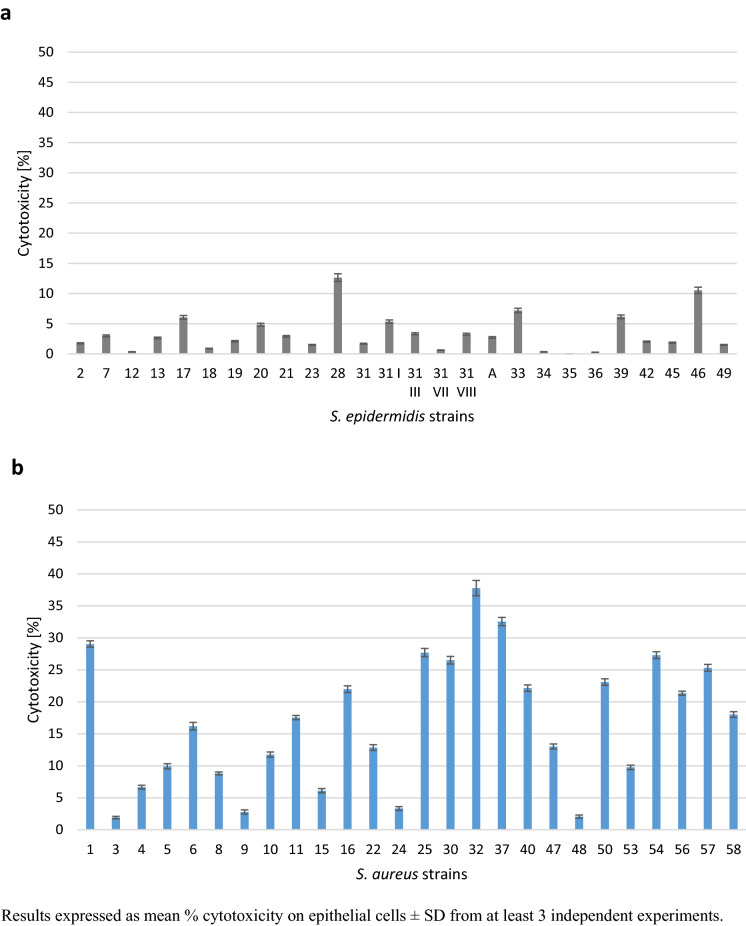
Cytotoxicity of different *S. aureus* and *S. epidermidis* strains at MOI 30 on epithelial cell line. **a** represents cytotoxic ability of *S. epidermidis* strains. **b** represents cytotoxic ability of *S. aureus* strains. Results expressed as mean % cytotoxicity on epithelial cells ± SD from at least 3 independent experiments

To investigate whether clinical isolates of SE and SA have the ability to kill host epithelial cells, their cytotoxic potential was evaluated in relation to Detroit cell line. The integrity of plasma membrane of host cells infected with MOI 30 of SA and SE clinical isolates was shown by measuring LDH released into the culture media 24 h post infection. The cytotoxic ability of SA strains to kill host epithelial cells was significantly higher than that observed in SE (*p* < 0.05; Fig. 5). As presented on Fig. 6b, most of the *S. aureus* strains (17 of 26 [65%]) proved to be able to kill epithelial cells after overnight incubation. But only a fraction of these isolates (11; 46%) produced significant (> 20% of host cells killed; p < 0.05) cytotoxicity 24 h after infection at MOI of 30. In contrast, SE strains showed weak cytotoxicity in relation to the host cells. Only in 3 cases (11.5%) cytotoxicity exceeded low 10% (Fig. 6a). Among highly toxic SA strains (11 strains no. 1, 16, 25, 30, 32, 37, 40, 50, 54, 56, 57) 6 (54.5%) were MDR (no. 25, 30, 50, 54, 56, 57), and they represented all MDR strains in a group of SA. Such a relationship was not noticed in the case of SE strains. Here, only one strain, no. 34, exhibited both weak cytotoxicity and MDR features.

## Discussion

For a majority of microorganisms, establishing an infection is a multifunctional process and different virulence factors can be involved in its pathogenesis. However, there is a correlation between bacterial strains isolated from specific diseases and the expression of specific virulence factors, which may indicate their special role in its pathogenesis [15]. An important feature of staphylococci, which has a great influence on the development of conjunctivitis, is the ability to adhere to artificial surfaces as well as to living cells and production of biofilm structures. It is crucial first step for the initiation of infection for the bacteria remaining in the colonized area. The area of the eyeball and conjunctiva is a place where there are a number of components that create a favourable environment for the development of bacteria, especially those producing large amounts of exogenous protein substances. Adherence and the formation of biofilm structures are important elements that affect the chronic, lasting even several weeks, character of conjunctivitis [15–17].

The results of our study are indicative of common adherence abilities demonstrated by staphylococcal strains involved in the aetiology of conjunctivitis as all tested isolates were capable of the biofilm development. Nevertheless, strong biofilm production mediated by the presence of a complete *ica* operon and subsequent production of a biologically active PIA evidenced by high absorbance values in the MTP assay was observed for only a minority of them.

The *ica* genes were detected by PCR in seven (26.9%) SE and in 12 (46.1%) SA isolates included in the study. Low prevalence of *ica* genes is staphyloccal isolates associated with ocular infections has also been reported in previous studies. Sharifinejad et al. observed that keratitis and endophthalmitis SE isolates showed the *icaA* + *, icaD* + *, is256* + gene profile more frequently (55.5% and 50%, respectively) than isolates cultured from the conjunctiva, nasolacrimal duct, and the eyelid for which the predominant (61%) profile was *icaA-, icaD-, is256-* [4]. Similarly, Flores-Páez et al. reported a low frequency predominance of 20–40% of *icaA, icaD* genes and IS256 distributed homogenously between identified sequence types of SE isolates cultured from the ocular infections including conjunctivitis and from the healthy conjunctiva samples (no statistical significance) [2].

In addition to relatively low level of *ica* genes detection, a high diversity of the PCR detected *ica* profiles was observed in the study reported here. It should also be highlighted that only four (7.6%) out of the 52 analysed isolates, represented only by SE, were both the *icaAD-* and *ica* operon-positive. The remaining *ica*-positive isolates were either *icaA* or *icaD*, or *icaAD*-positive and, interestingly, almost all of them (with the exception of one SA *icaD*-positive isolate) produced weak biofilm in the applied MTP assay. The results indicate that a complete set of four *icaADBC* genes arranged in a functional operon is the mainstay of the PIA-mediated biofilm formation.

Our results support the previous literature data on the necessity of the presence of a complete *ica* locus and the co-expression of *icaA*, *icaD*, and *icaC* for the functional PIA production [18]**.** Namely, three out of the four isolates reported as both *icaAD* and *ica* operon-positive were strong biofilm producers; another isolate with this gene profile was weakly adherent which, in turn, was indicative of low or even the lack of the expression of the PIA and suggested the alternative but much less efficient pathway of adherence. Piechota et al. who investigated the biofilm formation by SA strains isolated from hospitalized patients noted, in line with our results, that not all genes of the *ica* operon were detected in analysed strains and observed a statistically higher biofilm biomass in *icaADBC-* (51.5% of isolates) and *icaADB*-positive strains (26.1% of isolates) than in those which were only *icaAD*-positive (15.4% of isolates) [19]. Elkhashab et al., in turn, who investigated 50 staphylococcal strains isolated from conjunctival swabs reported, in contrast to our results, that only 60% and 50% of SA and coagulase-negative staphylococci (CNS) isolates, respectively, were the biofilm producers phenotypically but all biofilm-forming isolates were positive for the *icaA* gene. Only, two out of their isolates produced the biofilm phenotypically but were negative for the *icaA* gene [3].

The discrepancy between the *ica* operon and *icaAD* detection observed in our study has several possible explanations. Bacterial strains can have only gene segments, not the whole and functional *ica* operon. Arciola et al. observed phase variants of slime-producing SA and SE clinical isolates lacking the *icaA*, *icaD* segments and even the entire *ica* locus [9]. Also, mutations or insertions (for example, IS256) inactivating the *ica* genes and influencing the PCR result may occur [10, 18].

In spite of the fact that the PIA production is still considered the main mechanism of the biofilm formation in both SA and SE, alternative, PIA-independent forms of the biofilm production have also been reported, especially for SA [10]. As Arciola et al. noted, the observation that a minor proportion of SA strains can form biofilm even in the absence of the *ica* locus and that certain strains carrying such locus continue anyway to produce biofilm even after deletion of the locus is suggestive of existence of the *ica*-independent pathways [10].

Indeed, we observed that that seven (26.9%) SA and five (19.2%) SE isolates were identified as strong biofilm producers and for the majority of these isolates biofilm production was not dependent on the PIA, either due to the lack of the *ica* genes, or an incomplete *ica* profile. This observation was striking particularly for SA as none of the isolates was both *ica* operon and *icaAD*-positive. Also, as mentioned previously, only three SE isolates able to produce strong biofilm were positive for both the *ica* operon and *icaAD*. These isolates simultaneously developed the greatest biofilm biomass (OD > 0.5) which suggested that the PIA expression largely contributed to their proficient biofilm production.

The biofilm matrix is composed of extracellular polymeric substances other than PIA including e-DNA, teichoic acids, and proteins. The candidate proteins involved in the biofilm production include Bap (biofilm-associated protein) considered to play a prevalent role in human infections caused by coagulase-negative staphylococci, Aap (accumulation-associated protein) in SE, as well as SasG, SasC, protein A, and fibronectin-binding proteins: FnBPA, FnBPB for SA [10]. Mechanisms involved in the *ica*-independent biofilms are multifactorial. According to Figuerido et al., they are performed with different strain backgrounds and the major biofilm structural components produced by a specific strain might not be the same for all bacterial strains. At the same time, the same strain may have multiple mechanisms for biofilm formation, depending on the environmental signals. This ability might represent a strategy for survival in the multifaceted host environmental context [20].

Alternative mechanisms of the biofilm production probably concur and can be switched on in different phases of the infectious process, adapting the characteristics of the biofilm extracellular matrix in response to external stimuli, in order to colonize and establish the infection in host tissue [10, 20].

Since all staphylococcal isolates included in the study were capable of expression of their adherence abilities in the applied assay, it can be speculated that the PIA-independent mechanisms of the biofilm production do exist, and they are common among isolates associated with conjunctival infections. Also, in spite of the fact, that the PIA-independent biofilms tend to be weaker than those associated with the production of the polysaccharide [21], their production seems to be necessary and sufficient for the development of the infectious process in the conjunctiva.

Attachment of staphylococci to eukaryotic cells is a critical step to their pathogenesis. This initial attachment is dependent on bacterial cell wall-bound adhesins with the best documented role of MSCRAMMs. For many Gram-positive bacteria these surface proteins play a prominent role in the attachment and are strong primary factors required to facilitate the internalization process ([16, 22]. Specifically, for both SA and CoNS, the interaction between FnBP-fibronectin and integrin α5β1 on host cells is necessary and sufficient to promote bacterial adhesion and subsequent internalization [12, 16, 22].

The data of adhesion assay in which clinical isolates of SA and SE were in contact with host epithelial cells for 15 min revealed that SA isolates adhered more extensively to host cells than SE. This significant difference in the attachment ability between two tested Staphylococcal species seems to be caused by the type of host cells that were used for the experiment. As proved by Ridley et al., nasopharyngeal Detroit 562 cells have significantly lower fibronectin-binding capacity, which reflects α5β1 functionality on their surface. This fact resulted in relatively poor S. aureus adherence and invasion [22]. In our study, despite the limitations of Detroit cell line, SA adhered more extensively to the cells when compared to SE. This confirms that bacteria must use alternative mechanisms through which they become attached albeit at a lower level [17]. Among other attachment molecules, extracellular adherence protein (Eap), which is produced by all SA isolates but not by other staphylococcal species, was found to be important with respect to its adhesive functions, independent of the MSCRAMM-type adhesins [6, 16]. Also, initial adhesion of SE to host cells and its internalization can be mediated by FnBP-Fn-α5β1 integrin pathway independent mechanisms or molecules.

It should also be noted that although the adherence capabilities and the subsequent biofilm production are crucial for the initiation and maintaining the infection, other aspects of bacterial pathogenesis including antibiotic resistance and cytotoxicity must be taken into account to unveil the pathogenesis of this type of infection.

Cell invasion of non-professional phagocytes, including epithelial cells, contributes to the infection development. Infections caused by SA are typically associated with death of the host cell and the process that is induced by the cumulative action of different bacterial components [6]. The cytotoxic activity is mainly elicited by the secreted cytotoxins, in particular staphylococcal α-toxin. This major cytotoxic, pore-forming agent elaborated by SA, by inducing apoptosis causes cell membrane lysis and subsequently cell death. The toxin provokes apoptosis in a wide range of different mammalian cells and mediates ocular tissue damage [15]. The cytotoxic effect induced by bacteria is highly related to the virulence of the microorganism. Not surpassingly, in our study we observed that SA strains released higher level of LDH than SE strains, confirming the cytotoxic ability of *S. aureus* strains to kill host epithelial cells. Interestingly, 54.5% of SA strains that were highly cytotoxic were also multi-drug resistant. This observation is important for two reasons. First, infections caused by MRSA strains are difficult to treat and, in the case of ophthalmic infections, are rapidly progressing. Secondly, SA strains that are multi drug resistant, in particular MRSA, significantly influences the pathogenesis and pathology of conjunctival infections. Schlievert et al. analysed virulence of MRSA bacteria in terms of the toxin produced and found that the strains synthesized inflammatory cytolysins including α-toxin, δ-toxin, γ-toxin [23]. There is also an epidemiologic association of the SA MRSA (especially community acquired CA-MRSA) infections with production of PVL [15]. PVL is a cell-specific toxin able to lyse polymorphonuclear neutrophils. Even at low concentrations the toxin activates inflammatory response of neutrophils and thus contributes to pathogenesis of conjunctivitis [15]. Although the cell type that we used in our study is not a target for PVL, it must be taken into consideration that there may be more serious consequences from PVL—mediated lysis of host cells in response to SA MDR eye infection.

In our study SE showed less aggressiveness than SA. Indeed, SE as a commensal organism is adopted for the colonization of human skin rather than invasion. Even though it is involved in conjunctivitis, it should be regarded as an "accidental pathogen" responsible for chronic infections. Moreover, many of the factors important for commensal lifestyle of SE, such as the ability to form biofilm, are beneficial as virulence factors during infection [11, 12].

The mainstay of treatment for bacterial conjunctivitis is an empirical broad-spectrum antibiotic therapy. A significant obstacle, however, is the increasing resistance to first-line drugs among ocular, bacterial pathogens. Therefore, an important information characterizing the studied staphylococcal strains is the assessment of their drug resistance, especially in relation to the groups of drugs that are of the greatest importance in the treatment of conjunctivitis. The reported results indicate that antibiotic resistance is an important factor associated with the pathogenic potential of staphylococci associated with conjunctivitis, but we have not observed a correlation between antibiotic resistance and the *ica* gene profile or the phenotypic ability to produce biofilm. Methicillin resistance was detected in 34.6% SE and 26.9% SA isolates with 75% of the MR isolates found. However, a strong biofilm production was observed for only one MRSE and two MRSA isolates among which only MRSE harboured both the *icaAD* and *ica* operon correlating to the production of the functional PIA. The remaining MR staphylococci were identified as weak biofilm producers positive either for the *icaAD* (2 SE isolates) or *icaD* (4 SA isolates) only, or *ica*-negative (5 SE and 1 SA isolate). The lack of a significant correlation between susceptibility to methicillin and biofilm formation was also reported by Smith et al. who tested 972 clinical SA isolates [24]. Sharifinejad et al., in turn**,** investigating SE associated with ocular infections reported that 82.6% of isolates with the *mecA* gene corresponding to methicillin resistance simultaneously possessed the adhesion operon genes which stands in contrast to results obtained in our study [4]. Elkhashab et al. also found a significant association between the presence of the *icaA* gene in staphylococcal strains isolated from conjunctival swabs and multidrug resistance as 89.5% and 72.7% of the MDR SA and SE, respectively, were *icaA*-positive [3]. Similarly, Fariña et al. found widely distributed capacity of the biofilm production in clinical SE ocular isolates especially those which were *mecA* -positive and multidrug resistant. Biofilm and *mecA* were also detected more frequently in clinical SE than in those isolated from the healthy human conjunctiva (75% and 70% vs. 36.3% and 18.2%, respectively) [25]. Figueiredo et al., suggested, in turn, that the PIA type is more common in MSSA while *ica*-independent biofilms were more frequently observed in MRSA which can support the results obtained in our study as none of the MRSA isolates possessed a complete set of the *ica* genes [20].

## Conclusions

The results are indicative of complexity of factors involved in the biofilm production in staphylococci associated with conjunctivitis. Adherence abilities were commonly observed in staphylococci associated with conjunctivitis. However, low prevalence of isolates positive for a complete and functional *ica* locus and low prevalence of strong biofilm producers was detected. Existence of *ica*-independent mechanisms of biofilm production seem to be more common in SA. At the same time, SA has a larger arsenal of effective factors and mechanisms responsible for adherence to eukaryotic cells than SE. It is more virulent compared to SE, which was manifested by a significantly higher percentage of strains causing damage to the host cell structures.
